# Small-molecule polymerase inhibitor protects non-human primates from measles and reduces shedding

**DOI:** 10.1038/s41467-021-25497-4

**Published:** 2021-09-02

**Authors:** Kevin Wittwer, Danielle E. Anderson, Kristin Pfeffermann, Robert M. Cox, Josef D. Wolf, Sabine Santibanez, Annette Mankertz, Roland Plesker, Zachary M. Sticher, Alexander A. Kolkykhalov, Michael G. Natchus, Christian K. Pfaller, Richard K. Plemper, Veronika von Messling

**Affiliations:** 1 Veterinary Medicine Division, Paul-Ehrlich-Institute, Langen, Germany; 2 Programme in Emerging Infectious Diseases, Duke-NUS Medical School, Singapore, Singapore; 3 Institute for Biomedical Sciences, Georgia State University, Atlanta, GA USA; 4 WHO European Regional Reference Laboratory for Measles and Rubella, Robert Koch-Institute, Berlin, Germany; 5 Emory Institute for Drug Development, Emory University, Atlanta, GA USA; 6 Life Sciences Unit, Federal Ministry of Education and Research, Berlin, Germany

**Keywords:** Antivirals, Measles virus, Viral infection, Preclinical research

## Abstract

Measles virus (MeV) is a highly contagious pathogen that enters the human host via the respiratory route. Besides acute pathologies including fever, cough and the characteristic measles rash, the infection of lymphocytes leads to substantial immunosuppression that can exacerbate the outcome of infections with additional pathogens. Despite the availability of effective vaccine prophylaxis, measles outbreaks continue to occur worldwide. We demonstrate that prophylactic and post-exposure therapeutic treatment with an orally bioavailable small-molecule polymerase inhibitor, ERDRP-0519, prevents measles disease in squirrel monkeys (*Saimiri sciureus*). Treatment initiation at the onset of clinical signs reduced virus shedding, which may support outbreak control. Results show that this clinical candidate has the potential to alleviate clinical measles and augment measles virus eradication.

## Abstract (web summary)

Measles virus is highly contagious and outbreaks occur worldwide. Here the authors show that the orally bioavailable small-molecule polymerase inhibitor ERDRP-0519 prevents measles disease in squirrel monkeys and reduces virus shedding.

## Introduction

Measles virus (MeV) invades the body via the respiratory route and infects immune cells in the upper respiratory tract through the signaling lymphocytic activation molecule receptor SLAM/CD150. During an incubation period of ~10–14 days, infected cells home to mediastinal lymph nodes, where the virus infects resident SLAM/CD150^+^T- and B-cells^1,2^. This event results in peripheral blood mononuclear cell (PBMC)-associated viremia and is followed by viral spread to epithelial cells, which coincides with the onset of clinical signs including fever, conjunctivitis, and the measles-typical rash^2^. While many patients fully recover, mortality increases substantially in the presence of pre-existing conditions or malnutrition^3^, which led to over 200,000 measles virus-related deaths in 2019^4^. Prolonged immunosuppression in the aftermath of measles impairs memory responses to non-related infectious diseases^5^, compromising the health prospect of recoverees. Humans are the sole MeV reservoir and infection or vaccination usually result in long-lasting immunity, making the virus a candidate for eradication^6^. Despite major concerted efforts for two decades, a recent evaluation of global measles eradication concluded that the anticipated progress has not been made and previous gains on vaccine coverage were lost^4,7^. Past achievements are further challenged by the SARS-CoV-2 pandemic, which led to suspension of vaccination programs in developing countries, leaving 78 million children susceptible to measles^8^. Due to the exceptionally high contagiousness of MeV (*R*_0_ = 13.7–18)^9^, measles is typically among the first diseases to reemerge when vaccination coverage drops in an area^10^. Since 2017, and thus before COVID-19, lapsed vaccination coverage has resulted in measles reappearing in geographic regions that had previously been declared measles-free. These challenges create an urgent need for the development of effective pharmacological countermeasures that can consolidate progress towards global measles control and support vaccination-based eradication efforts^11^.

**Fig. 1:**
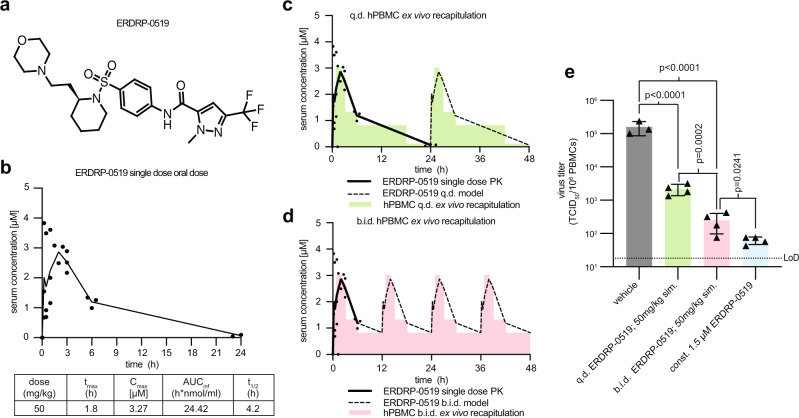
Single-dose oral pharmacokinetic study of ERDRP-0519 in squirrel monkeys. **a**, **b** ERDRP-0519 (**a**) was administered intragastrically at 50 mg/kg bodyweight in a poly(ethylene glycol)-200 formulation at a concentration of 10 mg/ml. ERDRP-0519 serum concentrations from blood samples were measured by liquid chromatography with tandem mass spectrometry (LC/MS/MS) (**b**); mean plasma concentrations are shown (*n* = 3). Pharmacokinetic parameters were calculated using the WinNonlin PK software package. C_max_ maximum calculated concentration, AUC_inf_ area under the curve extrapolated to infinity, t_1/2_ terminal elimination half-life. **c**, **d** PK-informed ex vivo assessment of anti-MeV efficacy in human peripheral mononuclear cells (hPBMCs) recapitulating once (*quaque die*; *q.d.*) or twice (*bis in die*; *b.i.d*) daily ERDRP-0519 dosing. Solid lines show the experimentally measured single-dose PK profile of the compound in squirrel monkeys; dashed lines represent the projected PK profiles, assuming no drug build-up after multi-dose administration; green and magenta blocks illustrate the experimentally applied concentrations in the 48-h ex vivo recapitulation of physiologically-achievable drug levels. **e** Progeny MeV titers observed in the ex vivo recapitulations shown in **c**, **d**. Statistical analysis through one-way ANOVA with Tukey’s multiple comparison post-hoc test. Symbols show individual biological repeats and bars illustrate the mean. *N* = 3 (vehicle) or *n* = 4 (all other groups), exact *p* values are stated, and error bars represent standard deviations; LoD limit of detection.

Previous studies have established high in vitro efficacy of the small-molecule viral polymerase inhibitor ERDRP-0519 (Fig. 1a) against MeV and related pathogens of the *Morbillivirus* genus, such as canine distemper virus (CDV)^12–14^. In vivo proof-of-concept efficacy was established in a lethal CDV-ferret surrogate model of human measles, demonstrating unprecedented complete survival of all treated animals and nearly complete suppression of clinical signs after post-exposure therapeutic dosing^13^. To assess the clinical potential of the compound, we evaluated in this study the oral efficacy against a clinical MeV isolate in non-human primates, which develop human measles-like disease.

## Results

### Efficacy of ERDRP-0519 against MeV at concentrations achieved in vivo

A single-dose pharmacokinetic (PK) study was performed in squirrel monkeys by oral (intragastric) delivery of 50 mg ERDRP-0519/kg bodyweight and blood sampling at seven predefined time points after dosing (Fig. 1b). Serum concentrations peaked ~2 h post-administration (*C*_max_ = 3.27 µM), which exceeded the in vitro EC_50_ values (0.07–0.3 µM, depending on the MeV-strain^13^) by 10- to more than 40-fold. Based on this PK profile, we recapitulated a once daily (*q.d.*) and twice daily (*b.i.d.*) treatment regimen ex vivo in human PBMCs infected with reference MeV strain MV/New Jersey.USA/94/1 (genotype D6) over a 48-h period, initially assuming no significant drug accumulation (Fig. 1c, d, Supplementary methods section, and Supplementary Table 3). Resulting progeny MeV titers were significantly (*p* < 0.0001) lower than in untreated controls (Fig. 1e). The *b.i.d.* dosing regimen furthermore showed significantly (*p* = 0.0002) higher potency than *q.d.* administration. Continuous exposure of MeV-infected hPBMCs to a conservatively estimated 1.5 µM ERDRP-0519 was nearly sterilizing, resulting in a greater than three orders of magnitude reduction in progeny virus titer (Fig. 1e). Informed by the ex vivo recapitulation of the antiviral effect of drug levels achievable after 50 mg/kg oral ERDRP-0519, we selected this dose level, given in a *b.i.d.* regimen, for the efficacy study.

### Clinical signs of measles virus infection in non-human primates are reduced under ERDRP-0519 treatment

**Fig. 2:**
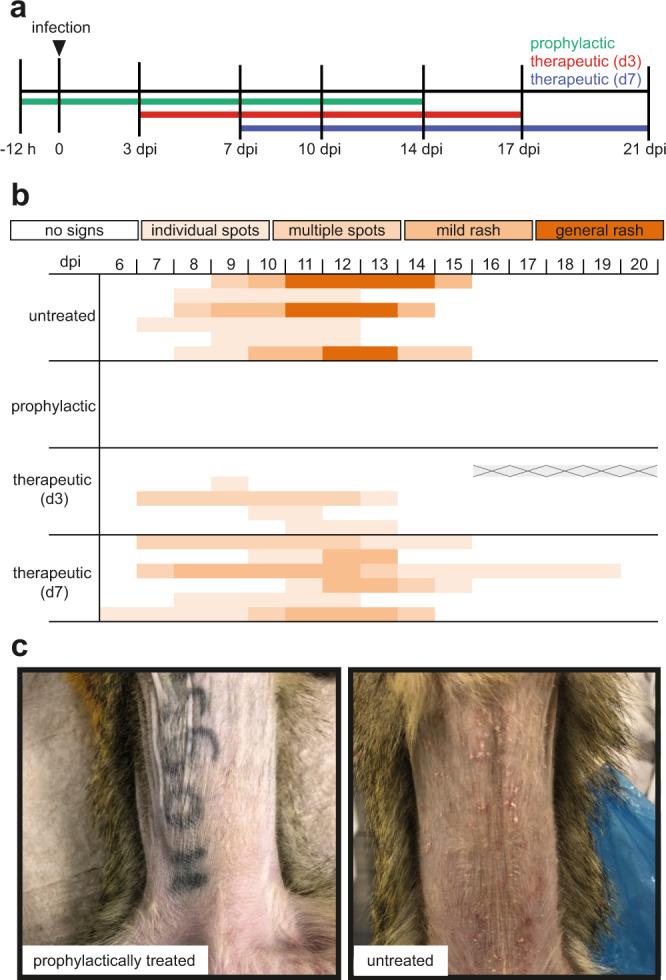
Effect of ERDRP-0519 on manifestation of clinical signs in MeV-infected squirrel monkeys. **a** Schematic illustration of the experimental design. Each group was treated twice daily (*b.i.d.*) over a 14-day period, illustrated by colored lines. Prophylactic treatment started 12 h prior to infection (green); therapeutic treatment started on day 3 post-infection (therapeutic (d3), red), or on day 7 post-infection (therapeutic (d7), blue). Dpi, days post-infection. **b** Clinical scores of infected animals. Each row represents one animal of the respective group and color intensity indicates severity of clinical signs categorized as no signs (white) or individual spots, multiple spots, mild rash, and general rash (light to dark graduation). Gray, crossed areas represent deceased animal. **c** Representative photographs of the abdomen of a prophylactically treated animal (left) and an untreated animal (right), taken 13 days post-infection.

To assess antiviral efficacy in vivo, squirrel monkeys were infected intranasally with 10^6^ TCID_50_ of the MeV field isolate MV/FrankfurtMain.DEU/17.11 (genotype D8), which has recently been highly prevalent in Europe^15^ and responsible for major measles outbreaks. A total of six animals per group were either left untreated, treated with ERDRP-0519 12 h prior to infection (prophylactic group), or treated 3 or 7 days after virus challenge (therapeutic groups), representing the onset of viremia and the appearance of first clinical signs, respectively (Fig. 2a). In all cases, treatment was continued for 14 days *b.i.d.* Untreated animals developed characteristic measles rash with inflammation around the mouth, nose and ears, and half of the monkeys in this control group presented a generalized rash (Fig. 2b, c, right panel). Prophylactically treated animals remained free of measles-typical clinical signs throughout the study (Fig. 2b, c, left panel). Four of six animals in the day 3 therapeutic group and all animals of the day 7 therapeutic group developed spots or mild rash in the face or inguinal region (Fig. 2b). However, none of the treated animals experienced severe or generalized rash, indicating a treatment benefit even when drug is administered late, at the time of onset of clinical signs.

### ERDRP-0519 treatment results in reduced viral replication and immunosuppression but efficient humoral response

**Fig. 3:**
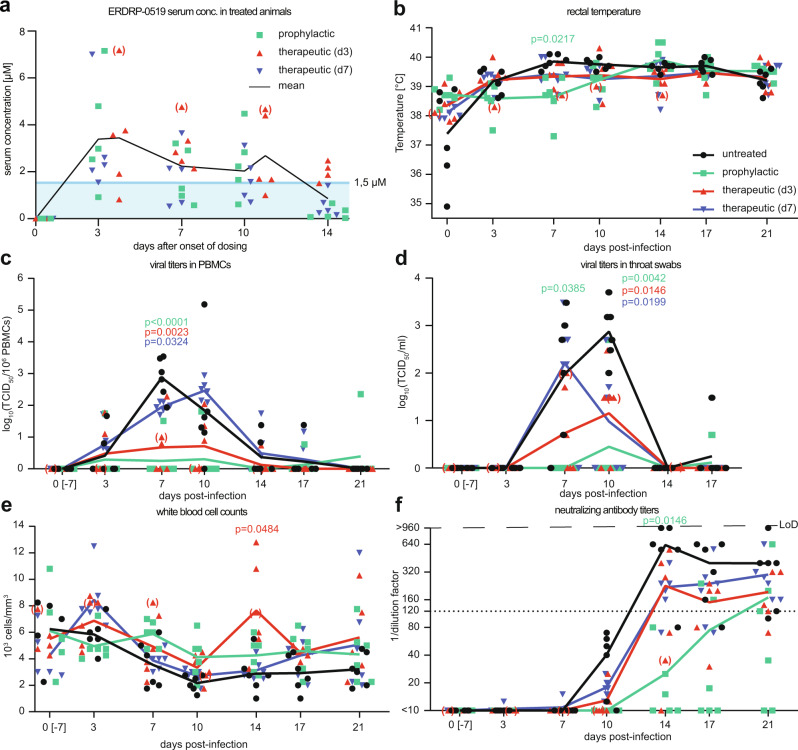
Clinical parameters of MeV infection in squirrel monkeys. **a** Trough serum concentrations of ERDRP-0519 in infected squirrel monkeys (*n* = 15) participating in this study followed over a 14-day *b.i.d.* oral treatment period. Solid black line shows mean values; blue line and square show the constant drug concentration (1.5 μM) applied in the ex vivo steady-state recapitulation to assess anti-MeV activity in hPBMCs shown in Fig. 1e. **b** Rectal temperature measured in the morning. **c**, **d** PBMC and throat swab titers, determined by end point dilution assay and expressed as log_10_(TCID_50_/10^6^ PBMCs) or log_10_(TCID_50_/ml) throat swab suspension, respectively. **e** White blood cell counts from whole blood, expressed as 10^3^ cells/mm³. **f** Neutralizing antibody titers, expressed as reciprocals of the highest serum dilution showing no signs of infection. Dotted line represents the expected protective antibody titer. LoD limit of detection. The day 0 blood samples and swabs were collected 7 days before the start of the experiment (0[-7] days post-infection). Statistical analysis with two-way ANOVA with Dunnett’s multiple comparisons post-hoc test was applied in **b**–**f**, using the untreated group as reference. Significant *p* values (*p* < 0.05) are shown, color-coded by group. For a *n* = 5 for all groups, for **b**–**f** *n* = 6 for all groups, except *n* = 5 for the 17 and 21 dpi time points in the therapeutic d3 group and *n* = 5 for the 0 dpi time points for Fig. 3e. Deceased animal is illustrated in parenthesis in all sub-panels; therapeutic (d3) and therapeutic (d7), therapeutically treated groups where treatment started on day 3 or day 7 post-infection, respectively.

Assessment of trough serum concentration during multi-dose *b.i.d.* administration of ERDRP-0519 in 15 animals confirmed that repeat-dosing does not result in ERDRP-0519 accumulation (Fig. 3a). Mean drug plasma concentration of all animals over the full study period was ~1.6 µM, and mean trough concentrations at individual time points exceeded the conservatively estimated 1.5 µM in a 12-day window after treatment start used for ex vivo hPMBC recapitulation experiments (Fig. 1e). Rash coincided with increases in body temperature in some of the animals but mean temperatures in the distinct groups remained within the pre-infection range of less than 40 °C (Fig. 3b). Body weight changes remained negligible in all animals throughout the study. One animal in the day 3 therapeutic group developed mild diarrhea and deceased on day 15 post-infection (illustrated in parenthesis). Viremia titers and throat swab titers in this monkey were in the lower range of animals in the group and leukocyte numbers were in the normal range. Drug serum concentrations of this animal were in the peak group of all treated animals (Fig. 1a), but none of the other monkeys with similar drug serum concentrations showed adverse effects. Nevertheless, to rule out toxic effects of the administered compound, we performed histological analysis of the liver of the deceased animal and found no pathological alternations when compared to an untreated control animal (Supplementary Fig. 1a–d). Furthermore, we examined the gut histologically since a potential, underlying parasitic infections may have worsen under stress of the study procedure. No multicellular parasites were found in the lumen of the deceased animal and no infiltration of eosinophilic granulocytes was detected, indicating absence of a parasitic infection (Supplementary Fig. 1e–h). We concluded that the isolated death was due to causes unrelated to ERDRP-0519 administration and/or MeV infection and continued the study unchanged.

Blood samples and throat swabs were collected twice weekly throughout the study (Fig. 2a) and viral load was determined. In untreated animals, PBMC-associated viremia titers peaked around day 7 before gradual resolution. Virus burden on day 7 post-infection was significantly higher in the untreated group than in the prophylactic treatment group (*p* < 0.0001) and the day 3 (*p* = 0.0023) and day 7 (*p* = 0.0324) therapeutic treatment groups, respectively. Prophylactic treatment with ERDRP-0519 decreased viremia titers on average by up to 250-fold compared to untreated animals, and day 3 therapeutic administration likewise effectively suppressed virus replication (Fig. 3c). However, initiation of treatment at day 7 post-infection had no significant effect on the height of maximum virus load, which was reached on day 10 post-infection in this group. These results illustrate that pre- or early post-exposure treatment prevents or strongly alleviates, respectively, PBMC infection. Direct comparison of serum ERDRP-0519 concentrations (Fig. 3a) and PBMC-associated virus titers (Fig. 3c) in the individual animals at day 7 post-infection revealed that the drug was similarly efficacious over a wide range of concentrations and that onset of treatment was indeed the essential parameter influencing viremia (Supplementary Fig. 2). Viral titers in PBMCs correlated with MeV *N* RNA, further underlining the inhibitory effect of ERDRP-0519 on viral replication (Supplementary Fig. 3a). Virus was cleared in general within 21 days. Throat swab titers of animals in all study groups resembled the PBMC-mediated viremia profile, except for the day 7 therapeutic group. Shed virus was first detectable 7 days after infection (Fig. 3d). Compared to untreated animals, shed virus titers in the day 7 therapeutic group dropped significantly (*p* = 0.0199) by two orders of magnitude within 72 h of treatment initiation (Fig. 3d), indicating that the duration and/or efficiency of virus transmission from an index case to social contacts may be reduced by the drug, even when treatment is first initiated after the onset of the measles rash.

Total white blood cell counts revealed typical MeV-induced leukopenia in untreated animals, which peaked on day 10 after infection and was followed by slow recovery (Fig. 3e). In contrast, no leukopenia was observed in the prophylactic and day 3 treatment groups. In the day 7 treatment group, we observed a trend for lower total white blood cell counts, but this was not significant, and cell counts increased more rapidly at later time points compared to the untreated group, making alleviated immunosuppression in all animals that had received ERDRP-0519 conceivable (Fig. 3e). Furthermore, animals of all groups including those prophylactically treated developed on average protective neutralizing antibody titers of ≥120 (Fig. 3f).

To assess clearance of MeV from the lymphatic system, MeV *N*-gene RNA levels were evaluated in lymph nodes of all animals 21 days post-infection and for the deceased animal at the day of its death. Whereas some of the untreated monkeys still showed high MeV *N*-gene RNA prevalence in lymph nodes, RNA levels were significantly decreased in the prophylactic (*p* = 0.0295) and therapeutic day 3 (*p* = 0.0346) dose groups, indicating accelerated virus clearance in these groups compared to the untreated group (Supplementary Fig. 3b).

### Safety of ERDRP-0519 treatment

**Fig. 4:**
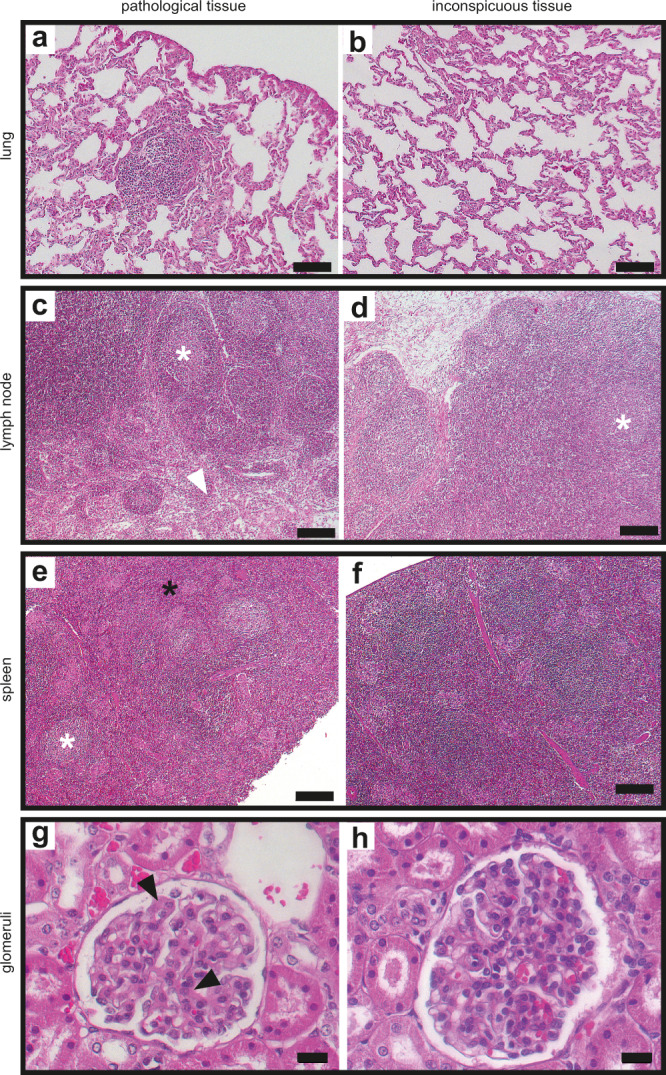
Histological examination on day 21 post-infection. H&E-stained tissues from all animals of the study (*n* = 24) were examined and representative pictures from individual animals are shown. **a** Perivascular lesion due to lymphocytic infiltration in the lung. **b** Normal lung tissue. **c** Lymph nodes with germinal centers (white asterisk) and histiocytosis (white arrow). **d** Normal lymph node. **e** Spleen with germinal centers (white asterisk) and hematopoiesis (black asterisk). **f** Normal spleen tissue. **g** Mesangial proliferation (black arrow) in glomeruli of the kidney. **h** Normal glomerulus. Black bar represents 100 µm (**a**, **b**), 200 µm (**c**–**f**), 20 µm (**g**, **h**). Representative pictures are shown from untreated group (**c**, **g**, **h**), therapeutic day 3 (**a**, **b**, **f**), or therapeutic day 7 (**d**, **e**) group.

All monkeys were sacrificed on day 21, followed by gross- and histopathological analyses. No histological signs of pharmacotoxicity were detected, but we noted mild histopathological changes such as lymphatic lesions in the lung (Fig. 4a, b), enlarged germinal centers in lymph nodes (Fig. 4c, d), hematopoiesis in spleen (Fig. 4e, f), and mesangial proliferation in glomeruli of the kidney (Fig. 4g, h), which are consistent with a recent virus infection. These and other manifestations emerged in a subset of animals of all treatment groups and thus cannot be linked to distinct treatment regimens (Supplementary Table 1).

Viral RNA was recovered from five animals per group at the last time point of viremia or positive swab titers for Sanger sequencing of the polymerase *L*-gene. No mutations causing amino acid changes were identified in the known clusters of the MeV polymerase protein associated with viral resistance to ERDRP-0519 in vitro^13,16^.

## Discussion

This study demonstrates the potential of ERDRP-0519 to improve management of severe measles and augment MeV eradication in primates. Our results show that mean attained compound serum titers in non-human primates after oral *b.i.d.* dosing were sufficient to significantly reduce MeV replication in an ex vivo recapitulation, anticipating that this dosing regimen inhibits viral replication in vivo. Indeed, we demonstrate a strong beneficial effect of ERDRP-0519 in MeV-infected squirrel monkeys leading to reduced clinical signs. Treatment started as late as 7 days post-infection prevented appearance of general rash. Earlier onsets of the treatment regimen further reduced disease severity.

ERDRP-0519 treatment reduced viral titers in PBMCs and throat swabs. Notably, prophylactic treatment nearly completely prevented MeV viremia, and treatment started 3 days post-infection reduced viremia markedly. In agreement with the intra-host life cycle of MeV^17,18^, treatment starting 7 days post-infection was unable to prevent PBMC-associated viremia, but still led to reduced viral titers in the throat of these animals, indicating decreased viral spread into and replication in epithelial tissues. The reduced viral load in airways may affect host-to-host transmission and thus have additional benefits in breaking outbreak chains.

Lymphocytopenia is a hallmark of MeV infection in humans, leading to immunosuppression during and after acute infection^5^. In our hands, animals treated with ERDRP-0519 trended to exhibit reduced immunosuppression compared to untreated animals, but this was not statistically significant. However, previous experiments of MeV-infected macaques had shown that elevated numbers of monocytes and eosinophils can compensate for MeV-induced lymphocytopenia^19^, indicating that a modest reduction in total white blood cells may be associated with an even more profound lymphocytopenia.

An important aspect of MeV infections in humans is the generation of life-long humoral immune responses that protect efficiently against re-infection. We demonstrate here that ERDRP-0519 treatment does not prevent the generation of humoral immune responses. All animals from the therapeutically treated groups developed high levels of neutralizing antibodies. In the prophylactically treated group, two animals did not develop neutralizing antibodies until the end of the study. It is possible that ERDRP-0519 treatment was so efficient in these individual animals that it prevented the minimal amount of viral replication required to mount efficient immune responses. However, PBMC-associated viral titers were similarly low in all animals of this group.

Absence of pathological alternations during necropsy and abnormalities that could be linked to a distinct group in histological examination suggests that ERDRP-0519 does not evoke toxic side effects at the administered dose, which is in line with previous in vivo experiments of ERDRP-0519 treatment in ferrets^13^.

The evolution of drug-resistant mutant viruses is a potential problem for the safety of any antiviral drug. Escape mutant hotspots occurring in the morbillivirus polymerase after ERDRP-0519 treatment have been characterized in cell culture and in vivo^13,16^. The relative fitness of these ERDRP-0519-resistant viruses was reduced compared to that of the drug-sensitive parent virus in all cases^13^, indicating that escape from the compound is associated with a selection disadvantage. Viruses re-isolated from ERDRP-0519-treated animals did not evolve resistance mutations, adding an additional positive aspect to the safety profile of the drug. Although it is challenging to predict the impact of emerging resistance on circulating MeV populations, the available datasets suggest that sustained circulation of ERDRP-0519-resistant MeV variants is unlikely.

Consistent with the devastating measles burden found in pediatric patients in many low- and middle-income countries, we believe that a viable therapeutic option must be amenable to cost-effective production, shelf-stable at ambient temperature, and orally bioavailable to allow administration independent of trained health care professionals^11^. The clinical candidate ERDRP-0519 meets these requirements, and is a therapeutic option for newborns and pregnant women not eligible to vaccination. In light of the current surge in MeV outbreaks in geographical regions with generally intermediate to high measles vaccine coverage^20^, an effective antiviral with the product profile of ERDRP-0519 could represent the critical missing link to ultimate global measles elimination, synergizing with vaccination by establishing a double-pronged prophylactic and post-exposure therapeutic anti-measles platform^21^.

## Methods

### Study design

All animal experiments were carried out in compliance with the regulations of German animal protection laws and authorized by the Regierungspräsidium Darmstadt, Germany (Dezernat V54 – Veterinärwesen und Verbraucherschutz, Hilpertstrasse 31, 64295 Darmstadt). Adult male and female squirrel monkeys (*Saimiri sciureus*) were obtained from BioPRIM, 31450 Baziege, or CNRS 0846 Primatologie, 13790 Rousset, France. Each experimental group was housed in a separate room, and animals of the same sex were caged together within the respective groups. For the single-dose pharmacokinetic experiment, animals were anesthetized, and the drug was given through a stomach tube at a concentration of 50 mg/kg bodyweight. Blood samples were collected after 15, 30, and 60 min, and 2, 3, 6, and 24 h. For efficacy assessment, groups of 6 animals were left untreated or treated with 50 mg/kg of ERDRP-0519 twice daily for two weeks, starting either 12 h before or 3 or 7 days after intranasal infection with 10^6^ TCID_50_ of MV/FrankfurtMain.DEU/17.11. Clinical signs, rectal temperature, and weight were recorded daily, and throat swabs and blood samples were collected before infection and twice weekly thereafter. On day 21 post-infection, the animals were euthanized, necropsied, and samples were collected for histological analysis.

### Cell culture and viruses

Vero cells stably expressing human SLAM (Vero/hSLAM) were maintained in Dulbecco’s modified Eagle’s medium (DMEM) with 5% fetal bovine serum and 1% l-Glutamine. For infection experiments, DMEM supplemented with 2% FBS and 1% l-Glutamine was used. Human peripheral blood mononuclear cells (iQ Biosciences; Donor 4327; Lot# P19L1400) were cultured in RPMI-1640 and stimulated with 0.2 μg/ml phytohaemagglutinin (PHA; Sigma–Aldrich) for 24 h prior to use. The isolate MV/FrankfurtMain.DEU/17.11 was used for infections of animals, and the isolate MeV/NewJersey.USA/94/1 was used for hPBMC infections. Each will be provided upon request.

### Compound synthesis and formulation

In total, 100 mg of ERDRP-0519^22^ was dissolved in 1 ml of poly(ethylene glycol)-200 (Sigma-Aldrich). On the days of treatment, the drug stock was diluted 1:10 in 0.5% methylcellulose under vigorous agitation to avoid precipitation. During all procedures, the drug was protected from light to avoid degradation.

### Pharmacokinetics

Drug concentrations were determined using an internal standard and a reversed phase isocratic HPLC method with positive ion electrospray ionization (ESI) mass spectrometry detection (LC/MS/MS) on an AB-SCIEX API 5500 MS/MS instrument (5 μl injection volume). Pharmacokinetic parameters were estimated using WinNonlin 5.3 (Pharsight).

### PK and PD recapitulation in human blood mononuclear cells

Human peripheral blood mononuclear cells (hPBMCs) were infected (MOI = 0.1 TCID_50_ units per cell) in a 24-well plate format with MeV/NewJersey.USA/94/1 (genotype D6) in the presence of ERDRP-0519 or DMSO (0.1%). The concentration of ERDRP-0519 was maintained at a constant 1.5 µM (recapitulation of trough steady-state drug levels achieved during *b.i.d.* dosing regimen in squirrel monkeys) or adjusted at various times post-infection to recapitulate ex vivo dynamic plasma drug concentrations for once (*q.d.*) or twice (*b.i.d.*) daily dosing regimens, based on single-dose pharmacokinetics in squirrel monkeys. Cells were collected 48 h after infection and virus was subsequently harvested after two freeze thaw cycles. Released virus titers were then determined by limited dilution method (TCID_50_) on Vero/hSLAM cells. Four biological repeats were used for each ex vivo PK recapitulation and three biological repeats were used for DMSO-treated hPBMCs.

### Virological and immunological sample analyses

White blood cell counts, viral load quantification in PBMCs, and quantification of neutralizing antibody titers were performed as follows: for white blood cell counts, blood was diluted 1:100 in 3% acetic acid and cells were counted using a Neubauer chamber. For viral load quantification in PBMCs, blood was centrifuged at 3000 rpm for 15 min and serum was frozen at −20 °C until further use. Red blood cell lysis was performed and the remaining PBMCs were counted using a Neubauer chamber. Viral titers in PBMCs were quantified in quadruplicates using limited dilution method and calculated as TCID_50_/10^6^ PBMCs. Neutralizing antibody titers were quantified by incubating MeV with 2-fold serial dilutions of the respective serum for 20 min at room temperature in quadruplicates. Vero/hSLAM cells were added and incubated for 3 days at 37 °C. Neutralizing antibody titers are expressed as the reciprocal of the highest serum dilution showing no signs of infection. For virus titration from throat swabs, swabs were placed in 150 μl DMEM with 3% penicillin/streptomycin and the titer was quantified by limited dilution method.

### Quantitative RT–PCR analysis of viral RNA in lymph nodes and PBMCs

Organs (21 days post-infection) and PBMCs (twice a week) were frozen in RNAlater (Qiagen) at −80 °C until further processing. For the deceased animal, organs were frozen in RNAlater at the day of death. Lymph nodes were thawed on ice, transferred to TRIzol (ambion life technologies) and homogenized using a tissue homogenizer. Total RNA of lymph nodes and PBMCs was extracted following the TRIzol manufacturer’s protocol. RNA (1000 ng for lymph nodes; 110 ng for PBMCs) was then subjected to reverse transcription using random hexamer primers and Superscript III reverse transcriptase in accordance with the manufacturer’s protocols. Subsequent quantitative PCR (qPCR) was carried out using primer pairs specific for a fragment in the MeV *N* gene^23^ or *GAPDH* gene(Supplementary Table 2) and the PowerUp SYBR Green Master Mix (Thermo Fisher Scientific) in an Applied Biosystems 7500 Real-Time PCR system. Samples were normalized for GAPDH. The comparative (ΔΔCt) Ct method was applied to determine relative amounts of N-encoding RNA present in samples from treated animals compared to those obtained from untreated control animals (lymph nodes) or compared to those obtained from untreated control animals on day 7 post-infection (peak PBMC virus titer).

### Genetic analysis

Viruses isolated from PBMCs and throat swabs were grown on Vero/hSLAM cells for one passage and RNA was isolated using the Direct-zol RNA MiniPrep Kit (Zymo Research) according to the manufacturer’s instructions. RNA was reverse transcribed using random hexamer primers and Superscript III reverse transcriptase and cDNA sequence was assessed by Sanger sequencing for presence of resistance mutations in clusters in the MeV *L* gene known to accumulate resistance-conferring mutations in tissue culture in the presence of ERDRP-0519^10^. Sanger sequencing was carried out using MeV *L* gene specific primers (Supplementary Table 2) and obtained sequences were analyzed using Sequencher software (Gene Codes Corporation; Version 5.4.6).

### Necropsy and histology

After euthanasia, each animal was examined for gross pathological changes, and samples were collected for histopathological examination. After fixation, all tissue samples were embedded in paraffin. Histology slices were mounted and stained using standard H&E staining techniques.

### Statistical analysis

Statistical analysis was performed using GraphPad Prism 8. One-way analysis of variance (ANOVA) with Tukey’s multiple comparison post-hoc test or two-way ANOVA, followed by Dunnett’s multiple comparisons post-hoc test were applied for statistical comparisons using the untreated group as reference if not stated otherwise. Different time points for each individual animal were paired. Biological repeat refers to measurements taken from distinct samples, and results obtained for each individual biological repeat are shown in the figures along with the exact size (*n* number) of biologically independent samples, animals, or independent experiments. Measure of center (connecting lines and columns) are means throughout. Error bars represent standard deviations (SD) throughout. For all experiments, the statistical significance level *α* was set to <0.05, exact *p* values (color-coded by experimental group) are shown in individual graphs wherever possible and significant.

### Reporting summary

Further information on research design is available in the Nature Research Reporting Summary linked to this article.

## Supplementary information

Supplementary Information

Reporting Summary

## Data Availability

All data that support the findings of this study are contained within the manuscript and the associated source data documents are provided in the supplement. Source data are provided with this paper.

## Source data

Source Data
