# Supplementary material for: Small-molecule polymerase inhibitor protects non-human primates from measles and reduces shedding
Source: Nat Commun. 2021 Sep 2;12:5233. doi: 10.1038/s41467-021-25497-4 (PMC8413292; doi:10.1038/s41467-021-25497-4)
Supplement: Supplementary file 1 — Supplementary Information [file 41467_2021_25497_MOESM1_ESM.pdf]

Supplementary Methods

PK and PD recapitulation in human blood mononuclear cells

Our PK analysis in squirrel monkeys revealed serum concentration fluctuations of ERDRP-0519 over a 24 h period after oral application (Fig. 1b). To test whether viral replication in peripheral blood mononuclear cells (PBMCs) was efficiently inhibited under these circumstances, we performed an *in vitro* recapitulation experiment. For this, we infected human PBMCs in a 24-well plate format with MeV/NewJersey.USA/94/1 (genotype D6) at a multiplicity of infection of 0.1 $TCID_{50}$ units per cell. To mimic the ERDRP-0519 serum concentration profile *in vitro*, we adjusted the drug concentrations in our PBMC cultures multiple times during the incubation period as shown by the colored areas in Fig. 1c (recapitulating a once per day treatment regimen, *q.d.*) and Fig. 1d (recapitulating a twice daily treatment regimen, *b.i.d.*) and summarized in Supplementary Table 3. The concentration changes were applied for a total of 48 h to the infected PBMC cultures (a total of two cycles of the *q.d.* regimen and four cycles of the *b.i.d.* regimen, assuming no drug accumulation during repeated treatment). As controls, infected PBMCs were incubated in the presence of vehicle only (0.1 % DMSO) or with a constant concentration of 1.5 µM of ERDRP-0519 over the whole period of 48 h post infection. Cells were collected 48 hours after infection and virus was subsequently harvested after two freeze thaw cycles. Released virus titers were then determined by limited dilution method ($TCID_{50}$) on Vero/hSLAM cells (Fig. 1e). Four biological repeats were used for each *ex vivo* PK recapitulation and three biological repeats were used for DMSO-treated hPBMCs.

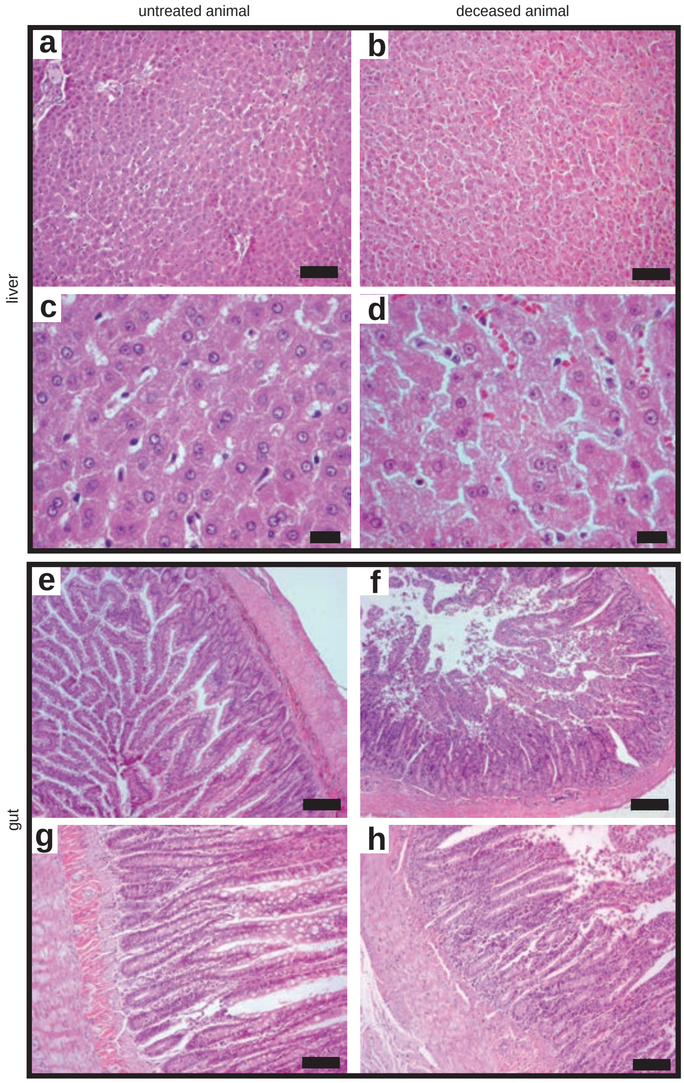

Supplementary Fig. 1: Histological examination of the deceased animal

Histological examination of H&E-stained liver and gut preparations was performed for the deceased animal and the untreated animals (n=6). Representative pictures are shown. a-d Liver screening revealed no pathological alternations in the deceased animal (b,d) compared to an untreated control animal (a,c). e-h gut examination for potential parasitic infection did neither reveal multicellular parasites, nor infiltration with eosinophilic granulocytes in the gut of the deceased animal (f,h) as compared to an untreated control animal (e,g). Scale bars represent 200 µm (e,f), 100 µm (a,b,g,h), or 20 µm (c,d).

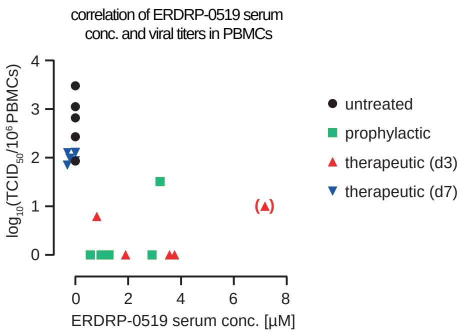

Supplementary Fig. 2: Correlation of ERDRP-0519 serum concentration and viremia

Correlation between serum concentration of ERDRP-0519 and PBMC titers of infected squirrel monkeys 7 days post-infection are shown. For each group n=5. Symbol in parenthesis indicates deceased animal.

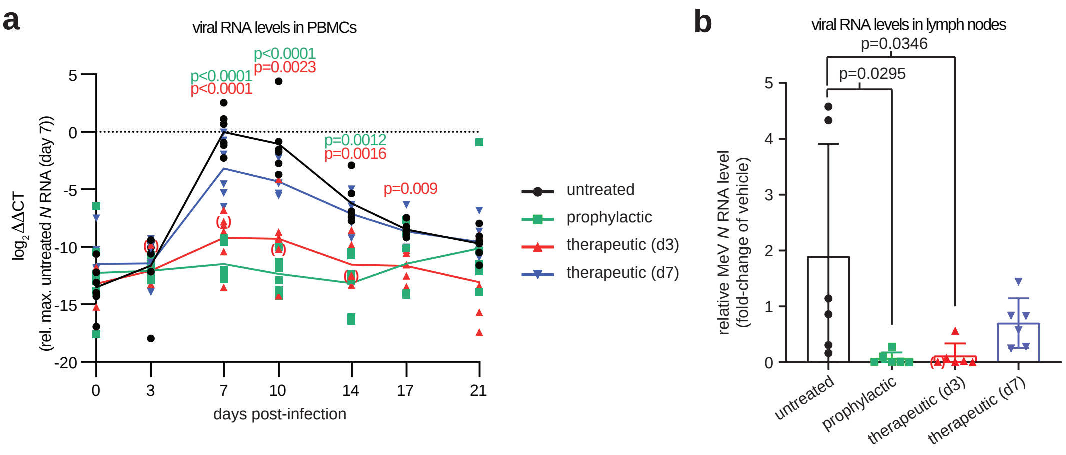

Supplementary Fig. 3: MeV *N* RNA in PBMCs and lymph nodes

a Relative MeV *N* RNA levels in PBMC compared to the mean of the untreated group 7 dpi (peak titers; dotted horizontal line). Lines indicate respective means at the individual time points. N=6 for all groups. Therapeutic (d3) and therapeutic (d7), therapeutically treated groups where treatment started on day 3 or day 7 post-infection, respectively. Deceased animal is illustrated by symbols in parenthesis. For statistical analysis two-way ANOVA with Dunnett‘s multiple comparisons post-hoc test was applied, using the untreated group as reference. **b** MeV *N* RNA in lymph nodes 21 dpi or in the case of the deceased animal at the day of death. N=6 for all groups. Statistical analysis through one-way ANOVA with Tukey’s multiple comparison post-hoc test. Bars represent mean values and error bars represent standard deviations. Symbols represent individual values and deceased animal is illustrated by symbol in parenthesis. P values are stated whenever significant ($p<0.05$).

Supplementary Table 1: Summary of events observed during histological examination of tissues from individual animals.

Group	Animal	Lung					Lymph node		Spleen			Glumeruli	
		NAD	Bronchitis/pneumonia	lymphatic	IP	granulomatous	NAD	alteration	NAD	White Pulp	Red Pulp	NAD	MP
untreated	1		B			G, GiC, FB	X			GC, CR	He		X
	2			L		G		GC		CR		X	
	3			L				n.d.		GC, CR			X
	4	X						CR, H		GC	He		X
	5		P					H		GC			X
	6	X						GC, H		GC	He	X	
prophylactic	1			L	IP	GiC, FB		GC, H		GC	He		X
	2		B	L	IP	G, GiC, FB		GC, H		GC		X	
	3				IP	GiC, FB		H		GC, CR			X
	4			L		GiC, FB		GC, H		GC	He		X
	5				IP	G, GiC, FB		n.d.			He		X
	6			L		GiC, FB		GC		GC		X	
therapeutic (d3)	1	X						GC		GC	He	X	
	2*				IP	G, FB		H		CR			X
	3				IP	FB		H		GC			X
	4		P					GC		GC			X
	5			L	IP	FB		GC,H		GC	He		X
	6	X						GC		GC	(He)	X	
therapeutic (d7)	1				IP			GC		GC, CR			X
	2		P	L	IP	GiC, FB		n.d.		GC, CR	He		X
	3			L	IP			CR, H		GC	He		X
	4		P					GC, H		GC, CR	He	X	
	5		P		IP	GiC, FB	X			GC, CR	He		X
	6			L				GC		GC		X	

NAD	no appreciable disease	P	Pneumonia	n.d.	not determined	*	deceased animal
IP	interstitial pneumonia	IP	interstitial pneumonia	CR	cell reduction		
GiC	giant cells	X	applicable	H	histiocytosis		
MP	mesangial proliferation	G	granuloma	He	hematopoesis		
L	lymphatic lesion	FB	foreign body				
B	Bronchitis	GC	germinal centers				

Supplementary Table 2: Primer sequences used for qPCR analysis and Sanger sequencing.

qPCR primers	
Primer name	Sequence
MeV N fwd 3	5'-TGGCATCTGAACTCGGTATCAC-3'
MeV N rev 3	5'-TGTCCTCAGTAGTATGCATTGCAA-3'
GAPDH fwd	5’-CCAAGGTCATCCATGACAAC-3'
GAPDH rev	5’-ACAGTCTTCTGGGTGGCAGT-3'
Sequencing primers	
Primer name	Sequence
MeV-L segment 1 fwd	5’-TCACTTGCTTACCTGCAGCTGAGGGAC-3'
MeV-L segment 1 rev	5’-TCAGGGTGATCAGTGTCTTGGTCCTGCC-3'
MeV-L segment 2 fwd	5’-TCCCGAAGCCCAGTCCACGCAAGTACC-3'
MeV-L segment 2 rev	5’-TGAGGTGATGGCCAATGTCGTGTAGCC-3
MeV-L segment 3 fwd	5’-TATGTGGGCAAGGCTAGCTCGGGGACG-3'

Supplementary Table 3: In vitro-recapitulation of ERDRP-0519 serum concentration fluctuations. The exposure times of MeV-infected PBMCs to indicated ERDRP-0519 concentrations over a single application cycle are shown recapitulating once daily (q.d.) or twice daily (b.i.d.) treatment regimens.

q.d. treatment regimen		*b.i.d.* treatment regimen	
ERDRP-0519 concentration [µM]	Exposure time [h]	ERDRP-0519 concentration [µM]	Exposure time [h]
2	1	2	1
3	1	3	1
2.7	1	2.7	1
1.3	3	1.3	3
0.8	12	0.8	6
0.1	6	-	-
