# Supplementary material for: Small-molecule polymerase inhibitor protects non-human primates from measles and reduces shedding
Source: Nat Commun. 2021 Sep 2;12:5233. doi: 10.1038/s41467-021-25497-4 (PMC8413292; doi:10.1038/s41467-021-25497-4)
Supplement: Supplementary file 2 — Reporting Summary [file 41467_2021_25497_MOESM2_ESM.pdf]

nature research

Corresponding author(s): Christian K. Pfaller; Richard K. Plemper

Last updated by author(s): Jul 30, 2021

Reporting Summary

Nature Research wishes to improve the reproducibility of the work that we publish. This form provides structure for consistency and transparency in reporting. For further information on Nature Research policies, see our Editorial Policies and the Editorial Policy Checklist.

Statistics

For all statistical analyses, confirm that the following items are present in the figure legend, table legend, main text, or Methods section.

n/a	Confirmed	
☐	☒	The exact sample size (*n*) for each experimental group/condition, given as a discrete number and unit of measurement
☐	☒	A statement on whether measurements were taken from distinct samples or whether the same sample was measured repeatedly
☐	☒	The statistical test(s) used AND whether they are one- or two-sided<br>*Only common tests should be described solely by name; describe more complex techniques in the Methods section.*
☐	☒	A description of all covariates tested
☐	☒	A description of any assumptions or corrections, such as tests of normality and adjustment for multiple comparisons
☐	☒	A full description of the statistical parameters including central tendency (e.g. means) or other basic estimates (e.g. regression coefficient) AND variation (e.g. standard deviation) or associated estimates of uncertainty (e.g. confidence intervals)
☐	☒	For null hypothesis testing, the test statistic (e.g. F, t, r) with confidence intervals, effect sizes, degrees of freedom and P value noted<br>*Give P values as exact values whenever suitable.*
☒	☐	For Bayesian analysis, information on the choice of priors and Markov chain Monte Carlo settings
☒	☐	For hierarchical and complex designs, identification of the appropriate level for tests and full reporting of outcomes
☒	☐	Estimates of effect sizes (e.g. Cohen's *d*, Pearson's *r*), indicating how they were calculated

Our web collection on statistics for biologists contains articles on many of the points above.

Software and code

Policy information about availability of computer code

Data collection	AB-SCIEX API 5500 MS/MS instrument<br>Applied Biosystems 7500 Real-Time PCR system
Data analysis	WinNonlin PK 5.3 (Pharsight)<br>GraphPad Prism Version 8.4.2<br>Sequencher software (Gene Code Corporation; Version 5.4.6)

For manuscripts utilizing custom algorithms or software that are central to the research but not yet described in published literature, software must be made available to editors and reviewers. We strongly encourage code deposition in a community repository (e.g. GitHub). See the Nature Research guidelines for submitting code & software for further information.

Data

Policy information about availability of data

All manuscripts must include a data availability statement. This statement should provide the following information, where applicable:

- Accession codes, unique identifiers, or web links for publicly available datasets
- A list of figures that have associated raw data
- A description of any restrictions on data availability

All data that support the findings of this study are contained within the manuscript and the associated source data documents provided in the supplement.
Source data and statistical analysis (if applicable) are available for:
Figure 1 b,e; Figure 3 a, b, c, d, e, f; Supp. Figure 2; Supp. Figure 3a, b

Field-specific reporting

Please select the one below that is the best fit for your research. If you are not sure, read the appropriate sections before making your selection.

- [x] Life sciences
- [ ] Behavioural & social sciences
- [ ] Ecological, evolutionary & environmental sciences

For a reference copy of the document with all sections, see nature.com/documents/nr-reporting-summary-flat.pdf

Life sciences study design

All studies must disclose on these points even when the disclosure is negative.

Sample size	Sample size was determined on previous experiences of MeV infection in squirrel monkeys; doi:10.1128/JVI.02490-16
Data exclusions	No data was excluded
Replication	- In vitro drug exposure experiment (Fig. 1e) was performed in three to four independent biological replicates.<br>- Single-dose pharmacokinetics data were obtained from three individual animals.<br>- Efficacy study: Initially, five animals per group were used for the efficacy study and assessment of ERDRP-0519 serum concentration. One additonal animal per group was subsequently treated/infected for the efficacy study.
Randomization	For in-vivo experiments, animals were randomly divided in groups representing balanced sexes of animals between the groups.
Blinding	Histopathology samples were blinded prior to scoring. No blinding occurred for any of the other experiments due to size of the research group. Individual experiments were carried out by a single investigator each and resources available did not allow involvement of additional personnel that would have been required for blinding.

Reporting for specific materials, systems and methods

We require information from authors about some types of materials, experimental systems and methods used in many studies. Here, indicate whether each material, system or method listed is relevant to your study. If you are not sure if a list item applies to your research, read the appropriate section before selecting a response.

Materials & experimental systems

n/a	Involved in the study	
x		Antibodies
	x	Eukaryotic cell lines
x		Palaeontology and archaeology
	x	Animals and other organisms
x		Human research participants
x		Clinical data
x		Dual use research of concern

Methods

n/a	Involved in the study	
x		ChIP-seq
x		Flow cytometry
x		MRI-based neuroimaging

Eukaryotic cell lines

Policy information about cell lines

Cell line source(s)	Vero/hSLAM - ECACC 04091501; doi: 10.1128/JVI.75.9.4399-4401.2001
Authentication	Vero/hSLAM cells were authenticated by G418 antibiotic resistance and by supporting measles virus replication.<br>Other than that, no specific techniques for authentication were performed.
Mycoplasma contamination	Cell lines were tested negative for mycoplasma contamination.
Commonly misidentified lines<br>(See ICLAC register)	No commonly misidentified cell lines were used in this study.

Animals and other organisms

Policy information about studies involving animals; ARRIVE guidelines recommended for reporting animal research

Laboratory animals	Species: Saimiri sciureus; sex: male and female; age: 1.5 - 17 years
Wild animals	no wild animals were used

Field-collected samples	no samples collected from the field were used
Ethics oversight	Dezernat V54 – Veterinärwesen und Verbraucherschutz<br>Hilpertstrasse 31<br>64295 Darmstadt<br>Regierungspräsidium Darmstadt, Germany

Note that full information on the approval of the study protocol must also be provided in the manuscript.
